# Inferring genome-wide patterns of admixture in Qataris using fifty-five ancestral populations

**DOI:** 10.1186/1471-2156-13-49

**Published:** 2012-06-26

**Authors:** Larsson Omberg, Jacqueline Salit, Neil Hackett, Jennifer Fuller, Rebecca Matthew, Lotfi Chouchane, Juan L Rodriguez-Flores, Carlos Bustamante, Ronald G Crystal, Jason G Mezey

**Affiliations:** 1 Department of Biological Statistics and Computational Biology, Cornell University, Ithaca, NY 14853, USA; 2 Department of Genetic Medicine, Weill Cornell Medical College, New York, NY 10021, USA; 3 Department of Genetic Medicine, Weill Cornell Medical College in Qatar, Doha, Qatar; 4 Department of Genetics, Stanford University School of Medicine, Stanford, CA 94305, USA

**Keywords:** Human migration, Admixture, Arabian Peninsula, Qatar, Support vector machines

## Abstract

**Background:**

Populations of the Arabian Peninsula have a complex genetic structure that reflects waves of migrations including the earliest human migrations from Africa and eastern Asia, migrations along ancient civilization trading routes and colonization history of recent centuries.

**Results:**

Here, we present a study of genome-wide admixture in this region, using 156 genotyped individuals from Qatar, a country located at the crossroads of these migration patterns. Since haplotypes of these individuals could have originated from many different populations across the world, we have developed a machine learning method "SupportMix" to infer loci-specific genomic ancestry when simultaneously analyzing many possible ancestral populations. Simulations show that SupportMix is not only more accurate than other popular admixture discovery tools but is the first admixture inference method that can efficiently scale for simultaneous analysis of 50-100 putative ancestral populations while being independent of prior demographic information.

**Conclusions:**

By simultaneously using the 55 world populations from the Human Genome Diversity Panel, SupportMix was able to extract the fine-scale ancestry of the Qatar population, providing many new observations concerning the ancestry of the region. For example, as well as recapitulating the three major sub-populations in Qatar, composed of mainly Arabic, Persian, and African ancestry, SupportMix additionally identifies the specific ancestry of the Persian group to populations sampled in Greater Persia rather than from China and the ancestry of the African group to sub-Saharan origin and not Southern African Bantu origin as previously thought.

## Background

The ancestry of people currently populating the Arabian Peninsula is complex. Centrally located among three continents, the geography of the region has contributed to migration influx during different epochs of human history. Qatar, located on the eastern edge of the Arabian Peninsula, is at the center of these patterns [1]. The first anatomically modern humans to leave Africa probably entered the peninsula around 125,000 years ago, followed by returning migration patterns of ancient humans from eastern Asia [2]. Qatar experienced seasonal migrations of early Arab tribes as trade flourished with ancient Mediterranean civilizations [1]. Before the start of the 20th century, the region was subjected to colonization efforts by the Portuguese, Ottomans, and British, and also saw an influx of Persian traders and African slaves brought through trade routes in Oman [3]. With increased prosperity in the last century, there has been an influx of traditionally nomadic populations into the cities and an influx of temporary guest workers from West and South Asia [3]. This rich migratory history has left traces in the genomes of individuals in the current population of Qatar. For example, a recent analysis of Qataris demonstrated three genetic sub-populations showing similarities to Arabic, Persian and African ancestry respectively [4]. Here, we perform a more detailed re-analysis of genetic structure in the Arabian Peninsula by analyzing genome-wide admixture in the modern population of Qatar.

Admixture in modern humans can reveal individual and population demographic histories and origins [5-7] and holds promise for determining the genetic basis for diseases [8-10]. The most basic historical scenario producing admixture is interbreeding of two ancestrally isolated populations over a small number of generations. For example, African-Americans in North America where the major ancestral populations are known because of the well-documented demographic history through the African diaspora and northern European migration in the 17th through 19th century [5]. The history of Qatar in contrast has produced a more complex pattern of admixture where not all ancestral populations are known [4]. An additional difficulty is there has not been extensive sampling in the region, so there is limited genetic data for the few ancestral populations that are known with greater certainty. Methods for analyzing broad-scale populations structure, such as principal components analysis (PCA) [11] and STRUCTURE [12,13] can be applied to such cases but lack in accuracy for loci-specific analysis [14]. Analyzing genome-wide admixture is a more significant challenge because available admixture inference methods cannot simultaneously analyze more than a few ancestral populations [5,15] or have poor performance on dense datasets, requiring filtering of SNPs by linkage disequilibrium [16-19]. Lack of complete knowledge of ancestral populations, due to poor sampling or the possible complete lack of well-defined ancestral populations [7], is therefore a roadblock to accurate genome-wide admixture analysis when applying these methods.

To address the challenge of inferring admixture for individuals from Qatar, we have developed "SupportMix," a machine learning method for admixture analysis. SupportMix is a two-level method where the first level makes use of support vector machines (SVM), a class of supervised machine learning algorithms known to be among the most accurate techniques for general classification problems, to identify the putative ancestral origin of a genomic segment. The second level is a smoothing application that makes use of a hidden Markov model (HMM) to detect transitions between ancestral origins in the admixed genomes (similar to [5,15,19]). SupportMix is an accurate approach for admixture analysis that can efficiently scale to genome-wide analysis when considering more than 50 ancestral population. In addition, because SupportMix is a data-driven learning approach, it is robust to specific population genetic model assumptions. When applying the method, it is therefore not necessary to make assumptions with regard to the underlying demography that lead to admixture. The combination of these properties means that SupportMix can be applied to genome-wide admixture analysis using a strategy that is impractical for other admixture methods. Instead of performing admixture analysis by determining a few ancestral populations and assuming specific demographic and population genetic parameters [5,15-19], with SupportMix, we can perform admixture analysis by simultaneously analyzing a large number of available world-wide populations as possible ancestors without being concerned with the relationship of these to the focal population. Given enough populations, SupportMix will return the genetically closest population to the ancestral population at each of the loci of the admixed genome. We also evaluate the accuracy of SupportMix by comparing the ancestry assignments to LAMP-ANC, a popular and accurate admixture method, [17] for a series of *in silico* generated admixed populations from the HGDP panel. By varying population genetic parameters we are able to infer the accuracy for many different admixture scenarios and show that not only is SupportMix more accurate but is also robust to perturbations in parameters.

We applied SupportMix to analyze admixture in 156 unrelated individuals from Qatar genotyped by microarray [4]. Since we are lacking complete knowledge of ancestry for the Qatar individuals, we used all 55 populations from the Human Genome Diversity Panel (HGDP) as possible ancestral populations [20]. SupportMix roughly divides the Qatari population into three sub-populations with different degrees of admixture, a result that corresponds well with both migratory history [3] and previous genetic studies [1,4]. One sub-population is of mainly Bedouin and other Arabic ancestry, "Arab-Qataris," one sub-population consists of admixture between Arabic and Greater Persian populations, "Persian-Qataris" and a third sub-population is heavily admixed between Arabic and African ancestry, "African-Qataris." The African alleles in the African-Qatari sub-population are most similar to northern sub-Saharan populations such as Yoruban, Mandinka and northern Bantu-speaking populations but dissimilar from southern Bantu populations, a result consistent with the historical slave trading routes. All three sub-populations show very low levels of admixture or similarity to European populations and some degree of admixture with Middle East populations from North Africa but levels of admixture below the error rate of other world populations. We compare the ancestry assignments of SupportMix to both principal component analysis (PCA) and STRUCTURE which both provide global predictions of ancestry proportions.

## Methods

We consider the problem of inferring the ancestral-population origin of the two phased haploid genomes of sampled individuals who can trace their recent ancestry to *k* genetically distinct populations. This is accomplished by training classifiers on *k*^*′ *^≥* k* putative ancestral populations where these *k*^*′ *^population must contain either the true *k* ancestral populations or good proxy populations for them. For each population multiple individuals have to be sampled at the same *N* polymorphic sites as the admixed individuals. Every ancestral and admixed haploid genome, *i*, we represent as a vector $x_{i}\in {\left\{-1,1\right\}}^{N}$ where -1 indicates the minor allele and 1 the major allele for the *N* sampled polymorphic sites. In addition every ancestral haploid genome is associated with a scalar *y*_*i *_∈ 1*..k*^*′ *^identifying the population of the haploid genome.

To find region-, or locus-specific ancestry assignments in admixed individuals, the vectors of genotypes, **x**_*i*_ are divided into windows, $x_{i}^{j}$, of *w* consecutive loci where *j* indexes the *N*/*w*windows in individual haploid genome *i*. The inference problem can therefore be formalized as finding the appropriate label $y_{i}^{j}$, for each window $x_{i}^{j}$, in an admixed individual *i*. We use support vector machines (SVM) [21] for this classification problem and apply a hidden Markov model (HMM) to the output of the SVMs to smooth the population assignment across windows.

### Training for ancestry classification with support vector machines

For each window of the genome we train a unique and independent SVM to optimize the classification of the *k*^*′ *^ancestral populations. For an excellent tutorial to SVMs we refer the reader to the article by Asa Ben-Hur [22]. Specifically a linear discriminant function is trained on each window, *j*, consisting of *w* markers. For the case *k*^*′ *^= 2 (more on *k*^*′ *^ > 2 below), the labels *y*_*i *_ become -1 and 1 and the discriminant function is found by the following constrained optimization problem:

$$\begin{array}{rl} \underset{w,b}{\min} & \frac{1}{2}∥w{∥}^{2}+C\overset{w}{\underset{i=1}{\sum }}\varepsilon_{i}\\ \text{subject to}: & y_{i}^{j}(w\cdot x_{i}^{j}+b)\geq 1-\varepsilon_{i},\;\varepsilon_{i}\geq 0\; \end{array}$$

where **w**is a vector and *b* a scalar that together define a *w*-1 dimensional hyperplane. This hyperplane optimally separates the two populations, in the *w* dimensional genotype space, subject to a penalty of misclassified individual samples proportional to the "slack" variable *C* and the distance, *ε*_*i*_, on the wrong side of the plane of sample *i*. The determined discriminant function $f(x_{i}^{j})=\text{sign}(w\cdot x_{i}^{j}+b)$ classifies an unknown genome window $x_{i}^{j}$ by determining $y_{i}^{j}$ of the window. For *k*^*′ *^ > 2 the standard one-against-one trick for binary classifiers is used, that is all *k*^*′*^(*k*^*′ *^− 1)/2 pairs of populations are trained against each other using the above standard binary SVM [23]. Then the unknown admixed window is tested using all the *k*^*′ *^(*k*^*′ *^− 1)/2 classifiers and the final ancestry is assigned the most common ancestry assignment of all these classifiers.

In addition to training the SVMs on the ancestral data, accuracy of the SVM classification is estimated with a three-way cross-validation. This was accomplished by subdividing the individuals in the ancestral populations into three independent sets, where SVMs were trained on two of the subsets and tested on the remaining third subset three different times. The accuracies of these three sub-samplings were averaged and taken as a measure of the success rate for each window across the *k*^*′ *^populations. This success rate is used heuristically to determine *w*, the size of the window necessary to discriminate the different ancestral populations while keeping *w* as small as possible to preclude recombination in the admixed genomes within each window and is also used as a parameter for the HMM.

### Application of SVMs for classification using hidden Markov models

The output classification of each admixed individual is used as the input to an HMM to extract the ancestral origins of each window. Each SVM is independent from every other SVM and as such, there is no assumed correlation between ancestral states across window boundaries. Biologically this correlation generally exists within genomic blocks as a consequence of linkage disequilibrium (LD). By introducing a Markov condition across windows, we reintroduce this correlation. Specifically the HMM has *k*^*′*^ hidden states and *k*^*′ *^output states. Assuming recombination occurs at every generation, recombination points are modeled along the chromosome as a Poisson process dependent on the recombination rate and number of generations, *g*, since admixture [24]. The transition probabilities between the hidden states is therefore modeled as (1 − e ^−* gd*^)/(*k*^*′ *^− 1) where *d* is the genetic distance between windows, measured in Morgans. The emission probabilities are *p* for the corresponding hidden state and (1−*p*)/(*k*^*′ *^− 1) for the other possible states, where *p* is the success rate of the SVM at the corresponding window as determined by the cross-validation (described in previous section). This HMM, in addition to accounting for LD, has the advantage of smoothing out the classifications returned by the SVM, so as to limit the effect of regions with poor information content. The posterior probability of the ancestry at each window location is determined using the forward-backward algorithm and the most probable ancestry is used as the estimated ancestry.

The genetic distance *d* between neighboring windows was estimated from the combined HapMap genetic map [25]. To test whether variations in the genetic map drastically affects the results a subset of simulations were repeated with a fixed *d* across the entire genome, where *d* was chosen to match the mean across the genetic map.

### Genotype samples from Qatar and the HGDP-Ceph panel

The 156 unrelated Qataris were processed on the Affymetrix 5.0 genotype array according to the protocols described by Hunter-Zinck et al. [4]. The 440,794 genotyped autosomal SNPs were pruned using PLINK v1.06 [26] to 327,044 SNPs with a minor allele frequency greater than 5%, a missing rate less than 5% and a Hardy-Weinberg equilibrium (HWE) deviation p-value of no less than 0.001.

The remaining SNPs were phased using Beagle [27]. For the examination of the Qatari sample using STRUCTURE the data was further pruned for pairwise linkage disequilibrium with a threshold of 0.5 in any 100 SNP window using PLINK's –indep-pairwise command and thinned to 20% of SNPs using –thin, resulting in 28,457 SNPs.

To assign segments of the genomes of each Qatar individual to possible ancestral populations, the HGDP sample data [20] was used. For these putative ancestral populations, we filtered for a minor allele frequency of 5% and removed related, unannotated and poor quality samples, retaining 886 individuals from 55 populations, all of which were phased using Beagle [27]. As the sample sizes of the different HGDP populations were very different the analysis was also repeated by subsampling 9 individuals from each HGDP population to verify that any results were robust to sample size changes. As the HGDP and the Qatar samples were run on different platforms only 71,982 SNPs remained for the analysis using SupportMix and PCA.

### Generation of in silico admixed population samples

A total of 651 unrelated individuals from 31 global populations in the HGDP-CEPH dataset that each have at least 10 sampled individuals (20 phased haploid genomes) were used for *in silico* analysis. The other HGDP populations were excluded as there were not enough individuals to simulate admixture and also exclude the ancestral indiviudals when training SupportMix. Admixed populations were generated by *in silico* mating of individuals from the different populations using a method similar to the one proposed by Price et al. [15] but extended to handle more than two ancestral populations. Specifically admixture between two ancestral populations in proportions *α *:(1 −* α*) was achieved by sampling *l* haploid chromosomes from each of the ancestral populations and mating each chromosome *i *∈ (1*..l*) with the corresponding chromosome in the other ancestral population using a recombination model. The origin of the admixed genomic regions was determined using two steps: 1. the initial origin was determined by probability *α* to be from population one, 2. the recombination spots along the genome were determined using a Poisson process such that the expected run lengths follow 1 −*e*^−*gd*^, where *g* is the number of generations since admixture and *d* is the distance along the genome measured in Morgans as determined by HapMap [25]. At each recombination site the originating genome was choosen with probabilities in the ratio *α* to (1 −* α*). For three or more populations, the admixture was determined by choosing the origin populations in ratios of *α*_1_:* α*_2 _:* α*_3 _:* ...*. For each admixed population, four haploid genomes were generated and the "ancestral" haploid genomes were discarded before the training of SupportMix and LAMP-ANC.

For admixture between two populations, 465 admixed populations were generated using all 31 ancestral populations with *α *= 0.5, *g *= 5. To explore the effects of *α*, *g*, window size and genetic map used, seven simulated admixed populations were used that spanned the range of population structure in these 465 populations. For three way admixture, mixture between Yoruban, French, and each of the other HGDP populations, as well as Yoruba, Bedouin and each of the other HGDP populations were simulated separately, with equal proportions for the populations (i.e. equal values of *α*) and *g *= 5.

### Comparison to other methods

SupportMix was compared to LAMP-ANC for *in silico* data and to PCA and STRUCTURE for the Qatar sample. The PCA was carried out on the 71,982 genotype SNPs common to both Qatar and HGDP using the singular value decomposition and STRUCTURE, using the built in admixture model [12], was run on the thinned-genotype dataset for *k *= 3 using 20,000 burn-in iterations and 10,000 iterations after burn-in. LAMP is a very accurate method for ancestral deconvolution of genotype data that has consistently been shown to do better than most previously published methods, while being able to handle more than two ancestral populations [17]. We did a quick comparison between LAMP-ANC and STRUCTURE run in the Linkage mode on in-silico generated African Americans. LAMP-ANC assigned 5% more loci correctly with the same number of loci used by both methods. LAMP is over an order of magnitude slower than SupportMix so to compare accuracy, a broad range of populations with different degrees of population structure were chosen. Specifically French-Bedouin, Bedouin-Yoruba, Han-Bedouin, French-Yoruba, Han-Yoruba, Papuan-Yoruba and Papuan-Karitiana admixture were examined with LAMP and SupportMix. LAMP was run in LAMP-ANC mode as described by Pasaniuc et al. [17].

## Results

### Accuracy of SupportMix assignments

**Figure 1:**
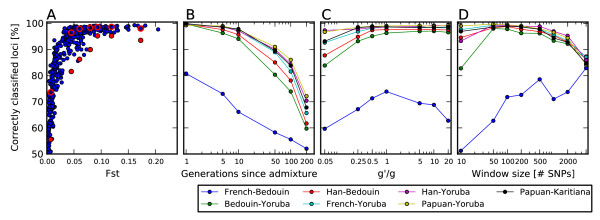
**Accuracy of SupportMix.** Accuracy of SupportMix ancestry assignments for *in silico* generated admixed populations. **(A)** Accuracy of ancestry assignment for two-way admixture between all HGDP populations (with more than 10 sampled individuals) using SupportMix (blue dots) and for a subset of 7 populations listed in legend using LAMP (red dots with the corresponding SupportMix results circled in red). Each *in silico* admixed population is plotted on the x-axis by the level of populations structure between the two ancestral populations as measured by fixation index, *F*_*st*_. **(B)** The effects of time since admixture, measured in generations, on SupportMix accuracy for 7 populations. **(C)** Effect on accuracy of uncertainty in generations since admixture, where *g*^*′ *^= 5 was the number of generations used to simulate the populations and *g* was the parameter used in SupportMix, which varied between .25 and 100 generations. **(D)** Effect of window size used on SupportMix accuracy of ancestry assignments.

**Figure 2:**
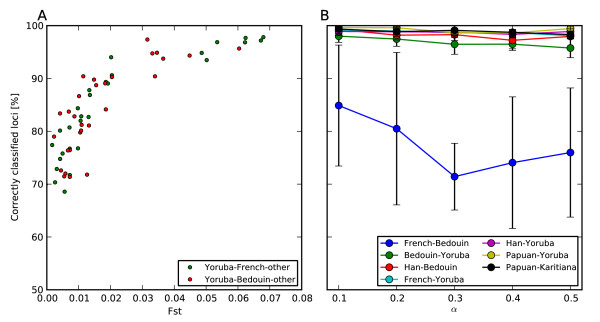
**Accuracy of SupportMix for three populations.** Accuracy of SupportMix ancestry assignments for *in silico* generated admixed populations. **(A)** Three way simulated admixture between Yoruba-French-other (green) and Yoruba-Bedouin-other (red) where "other”" is one of the remaining 29 HGDP populations with at least 10 sampled individuals. For each population the accuracy is plotted versus the lowest pairwise *F*_*st *_for the three ancestral populations. **(B)** Effects of different degrees of admixture proportions in two-way admixture, where *α*represents fraction of ancestry originating from the first population.

**Figure 3:**
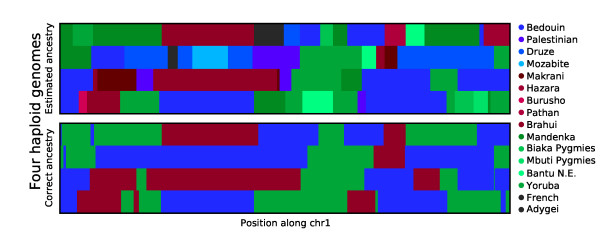
**In silico Qatari ancestry.** Ancestry assignments of two *in silico* generated Qatari individuals with equal proportions of Yoruba, Bedouin and Brahui ancestry. (top) Reconstructed ancestry assignment based on SupportMix using 31 HGDP populations and (bottom) true ancestry where colors represent the SupportMix assigned HGDP population as indicated by the legend on right side.

To assess the accuracy of SupportMix assigned ancestries in the Qataris, an analysis of *in silico* admixed populations was carried out where accuracy was measured by percent of correctly assigned loci. The accuracy was very high, especially for two populations with a high degree of population structure (Figure 1). Yoruba-French, *in silico* admixed individuals, for example, had 99.5% of the loci assigned correctly, which compares favorably with previously published methods of ancestry deconvolution using simulations between these same populations [15,17-19]. The accuracy was lower for more closely related populations but better than LAMP-ANC, a method that was been shown to consistently outperform other ancestry deconvolution methods by Pasaniuc et al. [17] (Figure 1). For three ancestral populations (*k *= 3) the accuracy of SupportMix was diminished slightly especially when two of the three populations were very similar, as determined by low *F*_*st*_ (Figure 2). To explore the effect of uncertainty in the ancestral populations we examined admixture between three populations using *k*^*′ *^= 31 in SupportMix. Specifically, we looked at a population similar to the Qataris, that is admixture between Yoruba, Bedouin and Brahui (Figure 3). Fifty percent of the loci were assigned to the exact correct population while simplifying the assignments into populations groups (Middle East, Greater Persia, sub-Saharan Africa and others) increased the accuracy to 87% (Figure 3).

For pairwise admixed populations, the effects of time since the start of admixture, fraction of ancestry, and perturbations were explored. Accuracy was lower for longer admixture periods with a significant drop in accuracy for more than 100 generations but no effect on ancestry fraction (Figures 1, 2). SupportMix has three free parameters: misclassification penalty, *C*, generations since start of admixture, *g*, and the window size, *w*. The misclassification penalty or "slack" parameter *C* which indicates the proportional penalty to assign to misclassified individuals during the training was varied from 1 to 1 × 10^5^ with no measurable change in accuracy. The HMM classification uses *g*, the number of generations since start of admixture, to determine the probability of recombination events occurring between windows. Using populations generated over 5 generations, *g* was varied between 0.025 and 100, a difference between 2.5 and 2000 years, assuming a generation length of 20 years. This showed that underestimating the time since admixture by 20-fold or overestimating by up-to 10-fold had little effect on accuracy (Figure 1). The optimal value for the third "window length" parameter is itself dependent on many variables: the time since admixture, the SNP density, and the population structure of the ancestral populations. Changes in one order of magnitude in *w* had little effect on the accuracy (Figure 1). When we fixed the recombination rate across the genome to 1.63cm/Mb instead of using the HapMap genetic map the accuracy was barely reduced seeing a maximum reduction of 0.9% for one of the 7 sampled populations (additional file 1).

### Ancestral origin of Qataris

**Figure 4:**
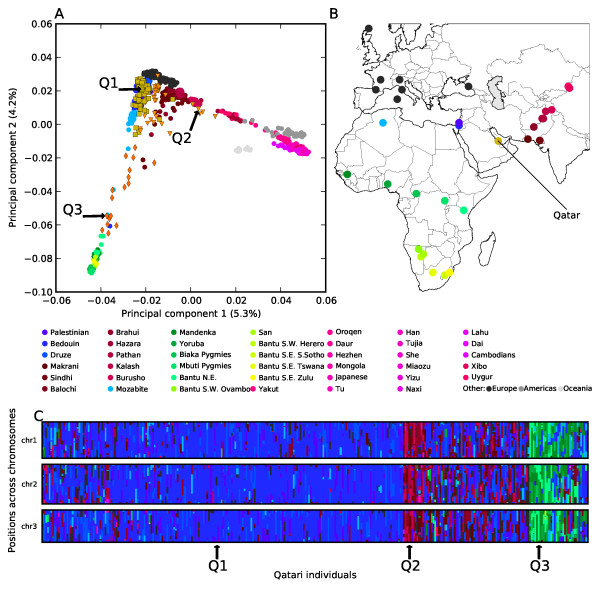
**Population structure of Qataris in comparison to HGDP world populations. (A)** Principal components 1 and 2 of all 55 HGDP populations along with Qataris colored by sampling origin. The Qataris, in orange, can roughly be divided into three sub-populations by general ancestry determined by PCA, STRUCTURE (see additional file 2) and SupportMix: squares, Arabic-Qataris; triangles, Persian-Qataris; diamonds, African-Qataris. Three sample individuals, one from each sub-population, are identified by arrows; Q1 from Arab-Qataris, Q2 from Persian-Qataris and Q3 from African-Qataris. **(B)** Sample locations of a subset of HGDP populations in region of world that showed significant similarity to the Qatari samples. Several European, Asian and all populations from the Americas and Oceania are outside the ploted region. **(C)** Locus-specific ancestry estimates of 156 Qatari individuals for chromosomes 1, 2 and 3 as determined by SupportMix. Each individual is shown as two vertical bars, one for each haploid genome where regions (from start of the chromosomes at the top to the end of chromosomes at bottom) are colored by the most similar ancestral HGDP population as indicated by color-legend and sample location in B. The three individuals in panel A are also indicated in panel C by arrows. Results of the entire autosomal genome are presented in additional file 2 along with estimates from STRUCTURE.

The admixture analysis of Qataris was carried out on unrelated individuals, all with four reported grandparents of Qatari descent. Using all 55 populations in the Human Genome Diversity Panel (HGDP) as putative ancestral populations, SupportMix roughly divided the Qataris into three sub-populations: Arab-Qataris, Persian-Qataris and African-Qataris, named after the origin of the majority of ancestral alleles. The sub-populations had different degrees of admixture between Middle-Eastern, Greater Persian and sub-Saharan African populations out of the 55 HGDP populations. This result was consistent, though more detailed than both STRUCTURE and principal component analysis (PCA) results (Figure 4 and additional file 2) repeated here, as in Hunter-Zinck et al. [4]. PCA and STRUCTURE were only able to show affinities toward either eastern Asian populations for the Persian-Qataris or affinities toward Bantu-speaking African populations for the African-Qataris but not the most similar ancestral populations [4].

**Figure 5:**
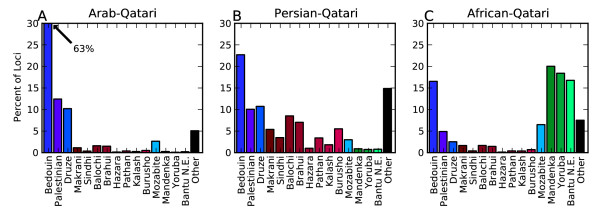
**Average Qatari ancestry.** Average proportion of loci assigned to each HGDP population by SupportMix in the three Qatari sub-populations: Arab-Qatari, Persian-Qatari and African-Qatari. The sub-populations were defined as in Hunter-Zinck et al. [4] using STRUCTURE and correspond to different degrees of admixture with **(A)** Arab-, **(B)** Persian- and **(C)** African-populations. Values are tabulated in additional file 3.

The Arab-Qataris showed very little admixture with non-Middle Eastern populations. Summarizing the assignments of loci by taking the mean number of loci per individual and the standard deviation across individuals, 88.5 ± 8.3% (expressed as mean ± standard deviation) of the loci were of Middle Eastern origin of which 63.2 ± 12.0% were most similar to Bedouins, 12.4 ± 5.3% to Palestinians and 10.2 ± 4.4% similar to Druze (Figure 5, additional file 3). In addition a small percentage of the loci (2.7 ± 2.5) were classified as stemming from the Mozabites, a Berber ethnic group in North Africa, also considered part of the Middle-East while other African populations were assigned less than 0.7% of the loci in the Arab-Qataris.

In contrast to the Arab-Qataris the two other sub-populations showed a large degree of admixture and variability in ancestral assignments, especially the Persian-Qataris. These individuals showed affinities toward eastern Asian populations in PCA space (Figure 4) [4] and had, in addition to Middle Eastern assigned loci (46.5 ± 18.1%), admixture with populations sampled in Pakistan and Afghanistan (36.2 ± 18.4%). There was a prevalence of alleles similar to the Balochi (8.5 ± 5.2%), an ethnic group that belongs to the Greater Persian peoples as well as the Brahui (7.0 ± 3.8%) that have mixed with the Greater Persian peoples and culturally resemble the Persians (Figure 5 and additonal file 2). Taking the sample location of these HGDP populations in Afghanistan and Pakistan, all within Greater Persia, and the lack of samples from Iran, it is reasonable to assume that these populations acted as proxies for Persian ancestry, as expected from historical migrations [3]. This is further strengthened by the fact that very few (< 1.5%) of the loci were assigned to South and East Asian populations, including Uygurs (< 0.2%) that had previously been suggested as showing affinity towards this sub-population [4]. In addition to Persian and Middle-Eastern ancestry, this sub-population had a few individuals with significant European ancestry (up to 72%), and across all individuals (12.9 ± 11.2%) of the loci were assigned to European populations, of which 2.7 ± 2.3% were assigned to Adygei, a European population located in the Caucasus at the border of Greater Persia (Figure 5 and additional files 2 and 3). While this high European component in some individuals could be due to recent European ancestry or remnants of European colonization, it could also be due to misclassification of a few individuals as not having Qatari ancestry or possible confusion by SupportMix because of genetic similarity of certain Persian populations with the HGDP European populations.

The third sub-population, the African-Qataris, showed strong admixture with African populations (60.8 ± 26.1%) and little admixture with the Persian populations (6.8 ± 7.8%) as well as fewer Middle-Eastern alleles (30.5 ± 20.7%) compared to the other two sub-populations. The African alleles were mainly assigned to populations in sub-Saharan Africa in the equatorial region, specifically, Mandenka (20.0 ± 9.0%), Yoruba (18.4 ± 10.5%) and Bantus sampled in Tanzania (16.8 ± 9.0%). There were very few (0.0 ± 0.3%) loci assigned to southern African populations such as the Bantu, Pygmy and San sampled in South Africa and Namibia (additional file 3). This is different from the results that were achievable using PCA, which could only show affinities toward Bantu populations but not the specific ancestral populations [4].

Across all three sub-populations virtually no loci were assigned to populations from the Americas, Oceania and eastern Asia (additional file 3). When verifying that the results were stable to variations sample sizes by only using a subset of 9 sampled genomes from each ancestral population the results changed very little; some of the African segments assigned Yoruba and Mandinka ancestry were reassigned to Tanzania Bantu ancestry while similarly some Bedouin tracts were reassigned to the other Middle Eastern populations, Palestinian and Druze.

## Discussion

The region of the modern country of Qatar has been at the crossroads of major migrations from the eras of ancient humans, early civilization and recent centuries [1]. The population of Qatar therefore provides a unique opportunity to study how the history of human migration is reflected in modern genomes. Using the new method, SupportMix, to study 156 Qatari individuals we have been able to infer the region-specific origin of the genomic segments of these individuals.

The SupportMix results are consistent with the migration patterns to Qatar prior to 1920 [3]. At a broad scale, the ancestry analysis confirms the genetic importance of three major populations who settled in the region and currently define three sub-populations in Qatar: Arabs, Persian migrants and African slaves [3]. The analysis also provides finer-scale resolution as to the specific ancestry of each genomic segment, allowing further statements concerning the origins of the Qatari people.

The Arab-Qatari ancestry was primarily assigned to Middle Eastern populations with the majority ancestry assigned Bedouin origins. Arab individuals in Qatar personally identify very strongly with either Bedouin-Arab ancestry or Hadar-Arab ancestry, where the latter refers to settled populations as opposed to the migratory nature of Bedouins [3]. This dichotomy of identification was not represented in the data however. Given that all three ancestral Middle Eastern populations included in the analysis were sampled in a geographically confined region, it is not necessarily surprising that it was not possible to discern this Bedouin versus non-Bedouin Arabic ancestry.

The Persian-Qatari sub-population likely traces back to the migrants who arrived from Persia after the great Persian famine in the late 1800's as well as Persian migrants with Arab roots, known as *Huwala* in Arabic [3]. This sub-population also has the highest degree of admixture in Qatar with other world populations, such as European and non Middle East Asian populations, as well as admixture with sub-Saharan African populations yet no evidence for strong admixture with eastern Asian populations such as the Uygurs. The European admixture seems to be isolated to a few individuals and might be evidence for very recent admixture and not representative of the whole sub-population.

The African-Qatari appear to be at least partially descended from African slaves brought to Qatar through Zanzibar and Oman before the 20th century [3]. While previous genetic analysis had shown a broad trend towards Bantu speaking populations [4], the local ancestry suggests a more western African population ancestry. This could be an indication of relative recent influx of African ancestry when the slave trade moved further inland from the eastern African coast. Another possibility could be the recent mixing with western African populations and North African populations, leading to similarities with other Middle Eastern populations of the most western African populations.

Overall, of the world-wide populations, only 10 populations (Bedouin, Palestinian, Druze, Balochi, Mozabite, Brahui, Madenka, Yoruba, Makrani and Bantu N.E.) contribute at least 2% of the ancestry to the Qatari population while the vast majority of the 55 world populations contribute less than 0.1% of the ancestry. Several regions, including those of African ancestry (Madenka, Yoruba, Bantu N.E. and Mozabite) and Persian ancestry (Balochi, Brahui, Makrani), are likely being assigned multiple ancestral origins because each acts as a proxy for a true, unsampled ancestral population.

## Conclusion

The Arabian peninsula is at the intersection of the ancient and historical migration patterns of three continents and the genomes of the people in the modern country of Qatar reflect this rich history. Our analysis of Qatari genomes has allowed us to infer the fine details of these migrations and to fill gaps in our understanding of the human migration history of this region. This analysis was made possible by SupportMix, which can make accurate assignments where prior specification of ancestral populations or a specific population genetic model is problematic to formulate. In a broader view, SupportMix provides a tool for accurate and robust ancestral assignment by simultaneous analysis of a worldwide selection of ancestral populations. Such analyses will be critical for accurate assignment in the many world-wide admixed populations that are likely to have unexpected ancestry that reflects a richer history than known from anthropological or historical studies.

## Author's contributions

LO drafted the manuscript, participated in the design of the study and concieved and performed the statistical analysis. JS, NH, RM and LC obtained samples and processed the genotyping data. JLRF aided in the researching the hisory of Qatar. CB conceived the initial idea of using machine learning to aid in ancestry assignments. JGM participated in the design of the study and together with RGC coordinated the project and helped draft the manuscript. All authors read and approved the final manuscript.

## Supplementary Material

Additional file 1 Table comparing accuracy of SupportMix using a genetic map versus a fixed recombination rate. Click here for file

Additional file 2 **Additional Figure 1 - Ancestry assignment for Qatari individuals for entire genome. Ancestry assignments for Qatari individuals.** (top) Locus-specific ancestry assignment from SupportMix colored by most probable ancestral population as in Figure 4 (shades of blue for Middle Eastern populations, reds for Asian populations and shades of green for African populations). Each individual haploid genome is represented by one column ordered top to bottom from the beginning of chromosome 1 to the end of chromosome 22. (bottom) Corresponding global ancestry assignment by STRUCTURE. Each individual (vertical bar) is colored by the proportion to the estimated ancestry in the *k *= 3 clusters. Click here for file

Additional file 3 Table of average ancestry assignments for the three Qatari sub-populations. Click here for file
