# Supplementary material for: Inferring genome-wide patterns of admixture in Qataris using fifty-five ancestral populations
Source: BMC Genet. 2012 Jun 26;13:49. doi: 10.1186/1471-2156-13-49 (PMC3512499; doi:10.1186/1471-2156-13-49)
Supplement: Additional file 1 — Table comparing accuracy of SupportMix using a genetic map versus a fixed recombination rate. [file 1471-2156-13-49-S1.pdf]

Table 1: Accuracy of SupportMix with different genetic maps

Ancestral Populations	HapMap genetic map	Constant Recombination rate
French, Bedouin	77.0%	76.1%
Bedouin, Yoruba	97.7%	97.7%
Han, Bedouin	97.8%	97.7%
French Yoruba	98.5%	98.3%
Han Yoruba	99.1%	98.9%
Papuan Yoruba	99.0%	99.0%
Papuan Karitiana	98.1%	98.2%
