# Supplementary material for: Inferring genome-wide patterns of admixture in Qataris using fifty-five ancestral populations
Source: BMC Genet. 2012 Jun 26;13:49. doi: 10.1186/1471-2156-13-49 (PMC3512499; doi:10.1186/1471-2156-13-49)
Supplement: Additional file 2 — Additional Figure 1 - Ancestry assignment for Qatari individuals for entire genome. Ancestry assignments for Qatari individuals. (top) Locus-specific ancestry assignment from SupportMix colored by most probable ancestral population as in Figure 4 (shades of blue for Middle Eastern populations, reds for Asian populations and shades of green for African populations). Each individual haploid genome is represented by one column ordered top to bottom from the beginning of chromosome 1 to the end of chromosome 22. (bottom) Corresponding global ancestry assignment by STRUCTURE. Each individual (vertical bar) is colored by the proportion to the estimated ancestry in the k = 3 clusters. [file 1471-2156-13-49-S2.pdf]

Additional Figure 1 - Ancestry assignment for Qatari individuals

Ancestry assignments for Qatari individuals. (top) Locus-specific ancestry assignment from SupportMix colored by most probable ancestral population as in Figure 1 (shades of blue for Middle Eastern populations, reds for Asian populations and shades of green for African populations). Each individual haploid genome is represented by one column ordered top to bottom from the beginning of chromosome 1 to the end of chromosome 22. (bottom) Corresponding global ancestry assignment by STRUCTURE. Each individual (vertical bar) is colored by the proportion to the estimated ancestry in the $k = 3$ clusters.

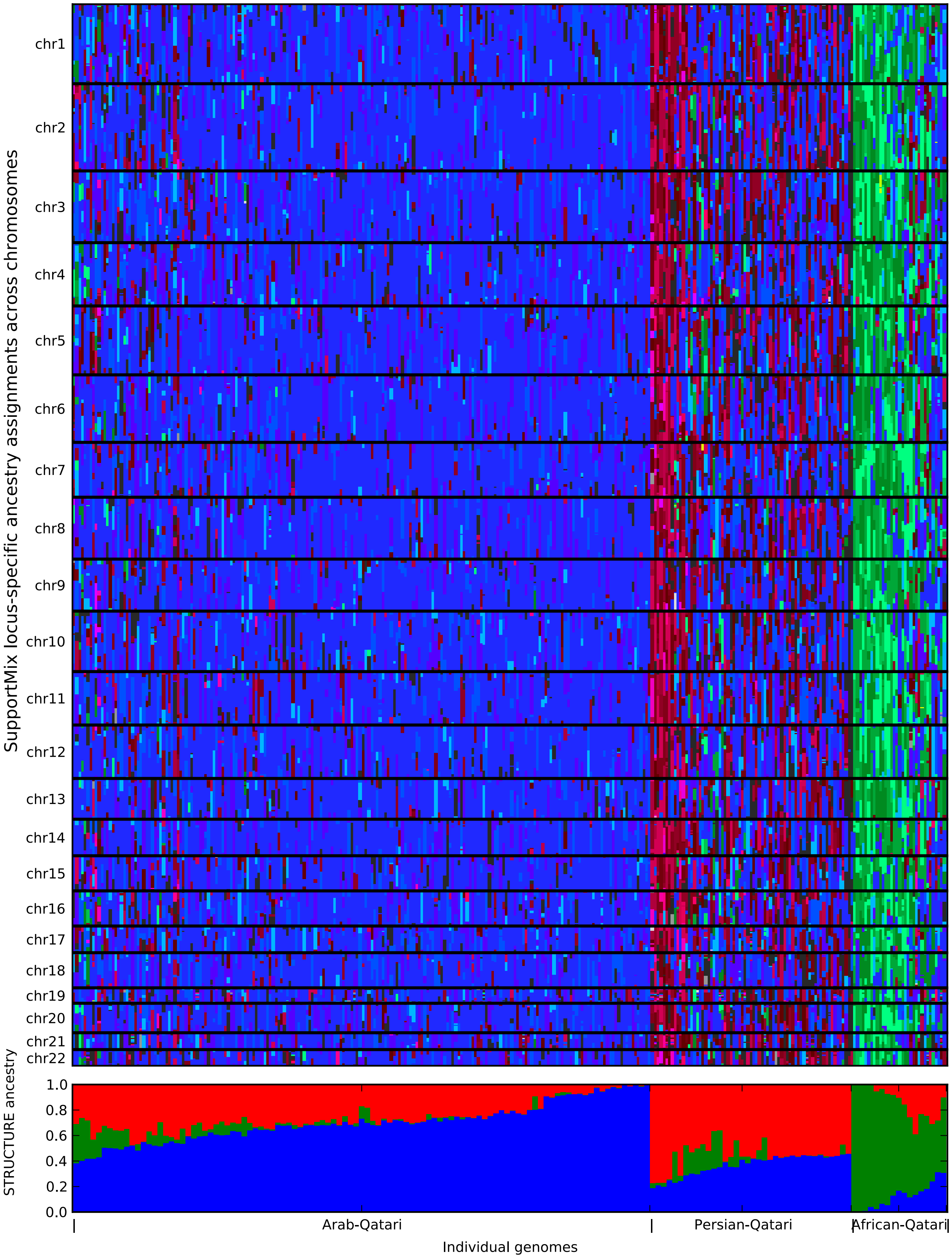
SupportMix locus-specific ancestry assignments across chromosomes
chr1
chr2
chr3
chr4
chr5
chr6
chr7
chr8
chr9
chr10
chr11
chr12
chr13
chr14
chr15
chr16
chr17
chr18
chr19
chr20
chr21
chr22
STRUCTURE ancestry
1.0
0.8
0.6
0.4
0.2
0.0
Arab-Qatari
Persian-Qatari
African-Qatari
Individual genomes
