# Supplementary material for: Inferring genome-wide patterns of admixture in Qataris using fifty-five ancestral populations
Source: BMC Genet. 2012 Jun 26;13:49. doi: 10.1186/1471-2156-13-49 (PMC3512499; doi:10.1186/1471-2156-13-49)
Supplement: Additional file 3 — Table of average ancestry assignments for the three Qatari sub-populations. [file 1471-2156-13-49-S3.pdf]

Table 2: Average proportion of ancestry assignments for the three Qatari sub-populations.

Region[1]	Population[2]	Arab-Qatari[%][3]	Persian-Qatari[%][3]	African-Qatari[%][3]
Middle East	Bedouin	63.2 ± 12.0	22.7 ± 10.8	16.5 ± 13.0
	Palestinian	12.4 ± 5.3	10.1 ± 5.6	4.9 ± 4.2
	Druze	10.2 ± 4.4	10.7 ± 5.9	2.5 ± 3.8
	Mozabite	2.7 ± 2.5	3.0 ± 3.0	6.5 ± 3.4
	All	88.5 ± 8.3	46.5 ± 18.1	30.5 ± 20.7
Greater Persia	Makrani	1.1 ± 1.4	5.4 ± 3.1	1.7 ± 1.9
	Sindhi	0.4 ± 0.8	3.5 ± 4.1	0.4 ± 0.7
	Balochi	1.6 ± 1.9	8.5 ± 5.2	1.7 ± 2.5
	Brahui	1.5 ± 1.8	7.0 ± 3.8	1.5 ± 2.0
	Hazara	0.1 ± 0.4	1.0 ± 1.1	0.1 ± 0.4
	Pathan	0.4 ± 1.0	3.4 ± 3.3	0.4 ± 0.9
	Kalash	0.3 ± 0.6	1.8 ± 2.0	0.4 ± 0.7
	Burusho	0.5 ± 0.9	5.5 ± 6.0	0.7 ± 1.1
	All	5.9 ± 5.4	36.2 ± 18.4	6.8 ± 7.8
sub-Saharan Africa	Mandenka	0.3 ± 0.9	0.9 ± 1.5	20.0 ± 9.0
	Yoruba	0.1 ± 0.4	0.7 ± 1.6	18.4 ± 10.5
	Biaka pygmies	0.1 ± 0.3	0.3 ± 0.7	5.3 ± 4.0
	Mbuti pygmies	0.0 ± 0.1	0.0 ± 0.2	0.3 ± 0.6
	Bantu n.e.	0.2 ± 0.7	0.8 ± 1.4	16.8 ± 9.1
	All	0.6 ± 1.8	2.7 ± 4.3	60.8 ± 26.1
southern Africa	All	0.0 ± 0.0	0.0 ± 0.1	0.0 ± 0.3
Europe	French	1.0 ± 1.3	3.4 ± 4.6	0.3 ± 0.6
	Russian	0.4 ± 0.6	1.5 ± 2.1	0.1 ± 0.2
	North italian	0.3 ± 0.6	0.6 ± 0.9	0.1 ± 0.2
	French basque	1.0 ± 1.3	2.0 ± 2.9	0.2 ± 0.5
	Adygei	0.9 ± 1.1	2.7 ± 2.3	0.3 ± 0.7
	Orcadian	0.2 ± 0.5	0.9 ± 2.0	0.1 ± 0.3
	Sardinian	1.0 ± 1.3	1.7 ± 1.6	0.3 ± 0.5
	Tuscan	0.1 ± 0.3	0.2 ± 0.5	0.1 ± 0.3
	All	4.8 ± 3.3	12.9 ± 11.2	1.5 ± 1.8
Asia	Cambodians	0.0 ± 0.3	0.2 ± 0.6	0.1 ± 0.4
	Han	0.0 ± 0.2	0.4 ± 1.2	0.1 ± 0.3
	Uygur	0.0 ± 0.1	0.2 ± 0.4	0.1 ± 0.3
	Yakut	0.0 ± 0.1	0.1 ± 0.4	0.0 ± 0.0
	All	0.2 ± 0.8	1.5 ± 3.0	0.3 ± 0.7
Other	All	0.0 ± 0.2	0.1 ± 0.4	0.1 ± 0.2

[1]Geographic sample location of ancestral population

[2]Ancestral population

[3]Mean $\pm$ standard deviation of fraction of loci across all individuals in a Qatari sub-population assigned to given ancestral population.
